# Gene Identification, expression analysis and molecular docking of ATP sulfurylase in the selenization pathway of *Cardamine hupingshanensis*

**DOI:** 10.1186/s12870-022-03872-7

**Published:** 2022-10-18

**Authors:** Zhijing Xiao, Yanke Lu, Yi Zou, Chi Zhang, Li Ding, Kai Luo, Qiaoyu Tang, Yifeng Zhou

**Affiliations:** 1 Hubei Key Laboratory of Biological Resources Protection and Utilization, Hubei Minzu University, 44500 Enshi, China; 2 College of Biological and Food Engineering, Hubei Minzu University, 44500 Enshi, China; 3 Hubei Minzu University Affiliated Enshi Clinical Medical School, The Central Hospital of Enshi Tujia and Miao Autonomous Prefecture, 445000 Enshi, Hubei China

**Keywords:** ATP sulfurylase, Selenium metabolism, Se hyperaccumulators, Molecular docking, Gene expression

## Abstract

**Background:**

ATP sulfurylase (ATPS) is a crucial enzyme for the selenate assimilation pathway in plants.

**Results:**

In this study, genome-wide and comparative analyses of ATPS in *Cardamine hupingshanensis*, including sequence and structural analyses, were performed. The expression of *ChATPS* gene family members in *C. hupingshanensis* under selenium (Se) stress was also investigated, and our results suggest that *ChATPS1-2* play key roles in the response to Se stress. Nine *ATPS* genes were found from *C. hupingshanensis*, which share highly conserved sequences with *ATPS* from *Arabidopsis thaliana*. In addition, we performed molecular docking of ATP sulfurylase in complex with compounds ATP, selenate, selenite, sulfate, and sulfite. ChAPS3-1 was found to have stronger binding energies with all compounds tested. Among these complexes, amino acid residues Arg, Gly, Ser, Glu, and Asn were commonly present.

**Conclusion:**

Our study reveals the molecular mechanism of *C. hupingshanensis* ATP sulfurylase interacting with selenate, which is essential for understanding selenium assimilation. This information will guide further studies on the function of the *ChATPS* gene family in the selenium stress response and lay the foundation for the selenium metabolic pathway in higher plants.

**Supplementary Information:**

The online version contains supplementary material available at10.1186/s12870-022-03872-7.

## Introduction

*Cardamine hupingshanensis*, also known as *Cardamine enshiensis*, is a unique selenium hyperaccumulator in China that can accumulate more than 1000 mg kg^-1^selenium [1]. *C. hupingshanensis* was first discovered in Yutangba, Enshi, Hubei Province, China, and Huping Mountain, Shimen, Hunan Province, China. Se hyperaccumulators growing in high-selenium environments for a long time have strong selenium tolerance, detoxification and enrichment abilities and have evolved unique molecular mechanisms. Therefore, Se hyperaccumulators have become an important resource for basic theoretical research on selenium. A chromosome-level genome assembly was performed for *C. enshiensis*, which consists of 443.4 Mb in 16 chromosomes with a scaffold N50 of 24 Mb [2]. Hi-C analysis of chromatin interaction patterns was performed, and genes with compartmental changes after selenium treatment were involved in the metabolism of selenium compounds [2]. Zhou et al. identified the biological pathways and candidate genes of the selenium tolerance mechanism by transcriptomics [3]. Differential expression analysis identified 25 genes in four pathways that are significantly responsive to selenite in *C. hupingshanensis* seedlings [3], including the ATPS genes and research targets in this paper.

Selenium is an essential element in humans and animals that plays a vital role in human health [4, 5]. Long-term severe selenium deficiency is the main cause of Keshan disease and Kashin-Beck disease [6]. Selenium deficiency increases the risk of cancer complications, and appropriate selenium supplementation can help reduce oxidative stress, thereby reducing the incidence of cancer complications [7]. In addition, an appropriate concentration of selenium plays an insulin-like role, but when the concentration of selenium is too high, it will aggravate insulin resistance and lead to type II diabetes [8]. Selenium mediates redox signalling and affects oxidative stress, inflammation and lipid metabolism and plays a certain role in improving the immune level of the human body, alleviating heavy metal toxicity, antiaging, preventing cardiovascular and cerebrovascular diseases, and relieving reproductive disorders [9]. In addition, recent studies have shown that the level of selenium in patients with COVID-19 is lower than that in healthy people [10]. Compared with selenium-deficient areas, selenium-enriched areas have a higher cure rate and lower mortality [11]. Moderate selenium supplementation may help prevent the deterioration of new coronary pneumonia patients [12].

Selenium is also considered to be a beneficial trace element for plants. Low doses of selenium can improve photosynthesis, promote plant growth [13–15], and contribute to the homeostasis of essential nutrients [16], while slightly higher concentrations are toxic. The distinction between selenium deficiency and selenium poisoning is very close, and because of this narrow gap, both selenium deficiency and selenium poisoning are widespread problems worldwide [17]. Although excessive accumulation of selenium can lead to phytotoxicity, low doses of selenium still have stimulatory effects on plants. Food sources of selenium are abundant, such as seafood, meat, cereals, vegetables, and nuts, while selenium from edible plants is a significant source of selenium for humans [18–20]. Therefore, researchers still hope that plants can accumulate selenium to restore the soil environment and alleviate the problem of selenium deficiency in selenium-deficient areas. Additionally, it can be used as a selenium supplement to assist in the treatment of diseases.

**Fig. 1:**
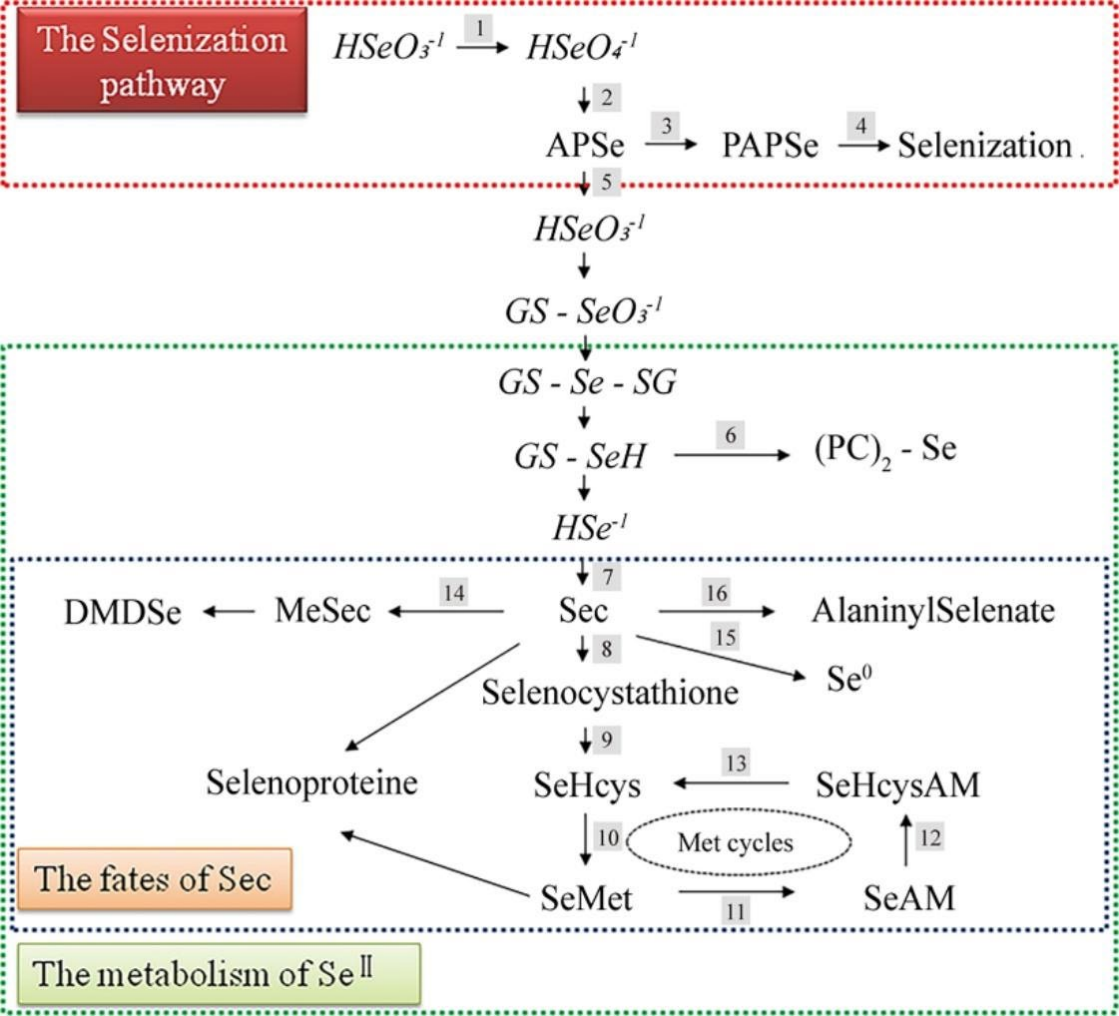
Diagram of plant selenium metabolism. Numbers denote known enzymes. (1) sulfite oxidase, (2) ATP sulfurylase, (3) adenosine phosphosulfate kinase, (4) sulfotransferase, (5) adenosine phosphosulfate reductase, (6) glutathione S-transferase, (7) cysteine synthase, (8) cystathionine-gamma-synthase, (9) cystathionine-beta-lyase, (10) methionine synthase, (11) S-adenosylmethionine synthetase, (11) SAM-dependent methyltransferase, (13) adenosylhomocysteinase, (14) selenocysteine methyltransferase, (15) NifS-like protein or selenocysteinelyase, (16) selenocysteinelyase. **Met cycles**: First, SeHcys may be converted to SeMet via methionine synthase, and SeMet is subsequently converted to SeAM by S-adenosylmethionine synthetase. Se-Adenosyl-L-selenomethionine (SeAM) is catalysed by SAM-dependent methyltransferase to generate Se-Adenosyl-L-selenohomocysteine (SeHcysAM), and then adenosylhomocysteinase catalyses the conversion of SeHcysAM to SeHcys

The metabolism of selenium in plants is mainly carried out through the metabolic pathway of sulfur in Fig. 1 [17, 21]. SeVI in plant roots is transported to leaf chloroplasts for metabolism, while SeIV can be metabolized in roots [21]. Excess SeIV can also be converted into SeVI by sulfite oxidase for metabolism [3]. Then, ATP sulfurylase (ATPS) catalyses the combination of selenate and ATP to form 5’-adenosine phosphoselenate (APSe) and release pyrophosphate (PPi) [1, 21]. When APSe is phosphorylated by adenosine phosphosulfate kinase (APK) to generate 3’phospho-adenosine-5’phosphoselenate (PAPSe), which provides a donor molecule for the selenylation of biomolecules, all possible hydroxyl groups of selenide molecules can be catalysed by cytoplasmic sulfotransferases [3]. When APSe is catalysed by adenosine phosphosulfate reductase (APR) to generate SeIV, SeIV can be combined with glutathione (GSH) to generate GS-SeO_3_^-1^, which is then combined with a molecule of GS-SeO_3_^-1^. The combined GSH generates GS-Se-SG, which is further reduced to GS-SeH and cleaved to HSe^-1^. Glutathione S-transferase promotes GS-SeH to form phytochelatins ((PC)_2_- Se) [22–24]. The cysteine synthase complex catalyses the synthesis of selenocysteine (Sec) from HSe^-1^ and O-acetylserine [25]. There are five metabolic paths for Sects. [26, 27]. (1) selenocystathione, selenohomocysteine (SeHcys) and selenomethionine (SeMet) are sequentially generated. (2) Methylselenocysteine (MeSec) is generated under the catalysis of selenocysteine methyltransferase. MeSec can be converted to dimethyldiselenide (DMDSe) by an as yet uncharacterized enzyme. (3) Zerovalent selenium is generated under the catalysis of NifS-like protein or selenocysteinelyase. (4) The SeH group of Sec is oxidized to generate alanine selenate or pyruvate selenate [3] or generate other water-soluble small molecules containing C-Se-C [28]. (5) Participation in the synthesis of selenoproteins or replacement of cysteine into proteins to form damaged or deformed selenoproteins, oxygen proteins and nitroproteins, which can be further removed by the proteasome [29, 30].

ATP sulfurylase is a key catalytic enzyme in selenium metabolism, acting as the first step in the metabolic pathway. The activation reaction of selenate, catalysed by ATPS to form APSe, is a rate-limiting step in the selenium metabolic pathway [31]. ATPS has been found in bacteria, fungi, algae and a variety of higher plants. ATPS plays an important role not only in sulfur metabolism but also in abiotic stress of various heavy metal ions [32, 33]. The catalytic substrate of SpATPS2 in *Stanleya pinnata* can be either sulfate or selenate, which can help plants accumulate selenium [34]. Experiments have shown that transgenic ATPS-overexpressing mustard plants accumulated more organic selenium and were more tolerant to selenium than wild-type mustard plants [35, 36]. Genome-wide identification of the ATP sulfurylase gene family has been conducted in many species [37–39], while few reports have focused on the functions of this gene family in *C. hupingshanensis*. Therefore, carrying out bioinformatics analysis of the key catalytic enzyme ATPS gene in the selenium metabolism pathway is preliminarily important to explore the mechanism of selenium accumulation and selenium tolerance in *C. hupingshanensis*.

Bioinformatics analysis of ATPS in 31 higher plants found that 84% of ATPS were located in the chloroplast, and the rest were located in the cytoplasm [38]. ATPS1-4 are found in *Arabidopsis*, mainly localized in the chloroplast [40], and selective translation enables *Arabidopsis* ATPS2 to be expressed in the cytoplasm as well [41, 42]. In plants, ATPS is a homodimer formed by the polymerization of two 48-kDa monomers [43]. Gene structure analysis ATPS contains 4–6 exons, and all ATPS contain the N-terminal domain PF14306 [PUA_2: PUA-like (pseudouridine synthase and archaeosine transglycosylase) domain] and C-terminal catalytic domain PF01747 (ATP-sulfurylase as catalytic domain) [38]. X-crystal diffraction analysis of soybean ATPS revealed that Arg248, Asn249, His255, and Arg349 play important roles in the enzymatic transition state [43]. ATP sulfurylase contains two highly conserved motifs: the HXXH motif and PP-loop [43, 44]. They contain several highly conserved histidine and arginine residues, all of which have functional side chains. ATPS exists in both allosteric and nonallosteric forms [45].

In this study, we aimed to screen and identify the substrate affinity of major members of the *C. hupingshanensis* ATPS family that respond to selenite stress genome-wide. First, we identified and analysed ATPS genes in *C. hupingshanensis* using a bioinformatic approach. The protein domain, gene structure, conserved protein motif and evolutionary tree of *C. hupingshanensis* ATPS gene family members were analysed to clarify the physicochemical properties and basic functions of *C. hupingshanensis* ATPS members. Second, qRT-PCR was used to screen the main gene from the ATPS family of *C. hupingshanensis* that reacted to selenite stress. Finally, molecular docking simulations were used to investigate the affinity between ATPS and the substrate.

## Results

### Identification and analysis of ATPS genes in C. hupingshanensis

We used the protein sequence of *Arabidopsis ATPS* in the *C. hupingshanensis* genome file to search the *C. hupingshanensis* genome file (the Genome Warehouse BIG Data Center accession number PRJCA005533) with BLASTp to identify potential *ChATPS* genes in *C. hupingshanensis*. Nine *ChATPS* gene family members were identified from the genome of *C. hupingshanensis*. They were designated ChATPS1-1 to ChATPS4-2 according to the homologous AtATPS. Detailed information about each gene, including gene name, nucleotide length, isoelectric point, predicted protein molecular weight and protein subcellular localization, is given in Table 1 and S1.

**Table 1:** The physicochemical properties of ATPS proteins in *C. hupingshanensis*

Gene ID		Gene name		Group		DNA Length (bp)	Intron #	Mature Protein (aa)	pI	MW (Da)	Subcellular localization prediction
*Chu048667*	*ChATPS1-1*		G1		1395		4	464	6.69	51377.95	Cytoplasmic
*Chu021913*	*ChATPS1-2*		G1		1389		4	462	6.48	51071.55	Chloroplast
*Chu001696*	*ChATPS2-1*		G2		618		4	180	7.02	20149.25	Cytoplasmic
*Chu015372*	*ChATPS2-2*		G2		1296		4	431	5.8	48,255	Chloroplast
*Chu015373*	*ChATPS2-3*		G2		1473		4	490	6.29	54720.22	Chloroplast
*Chu019428*	*ChATPS3-1*		G3		1398		4	465	8.35	52231.1	Chloroplast
*Chu028987*	*ChATPS3-2*		G3		1398		4	465	7.81	52086.02	Chloroplast
*Chu038431*	*ChATPS4-1*		G4		1398		4	465	8.61	52049.54	Chloroplast
*Chu044686*	*ChATPS4-2*		G4		1386		4	461	6.48	51526.91	Chloroplast

As illustrated in Table 1, the length of the nucleotide sequences of the identified ChATPS genes ranged from 618 to 1473 base pairs. All of these genes contain 4 introns. The protein sizes of ChATPS members ranged from 180 aa (ChATPS 2 − 1) to 490 aa (ChATPS 2–3). Accordingly, the MW of ChATPS members spanned from 20149.25 Da to 54720.22 Da. In addition, the theoretical isoelectric points of the ChATPS members ranged from 5.8 to 8.61. As predicted by the online servers SignalP-5.0 and TMHMM-2.0, all ChATPS proteins have no signal peptide and no transmembrane region. We predicted the subcellular localization of the protein by aligning with the N-terminal mature peptide homologous sequences of chloroplast ATP sulfurylases from *Spinach* and *Arabidopsis*, combined with an online server [40, 46, 47]. Preliminary prediction of the subcellular locations of the members showed that ChATPS members are located in the extracellular region, two of which are located in the cytoplasmic region, and the remaining 7 are located in the chloroplast. The gene coding sequences and protein sequences of the ChATPS family members are listed in Table S2.

### Phylogenetic analysis of ATPS genes in C. hupingshanensis

**Fig. 2:**
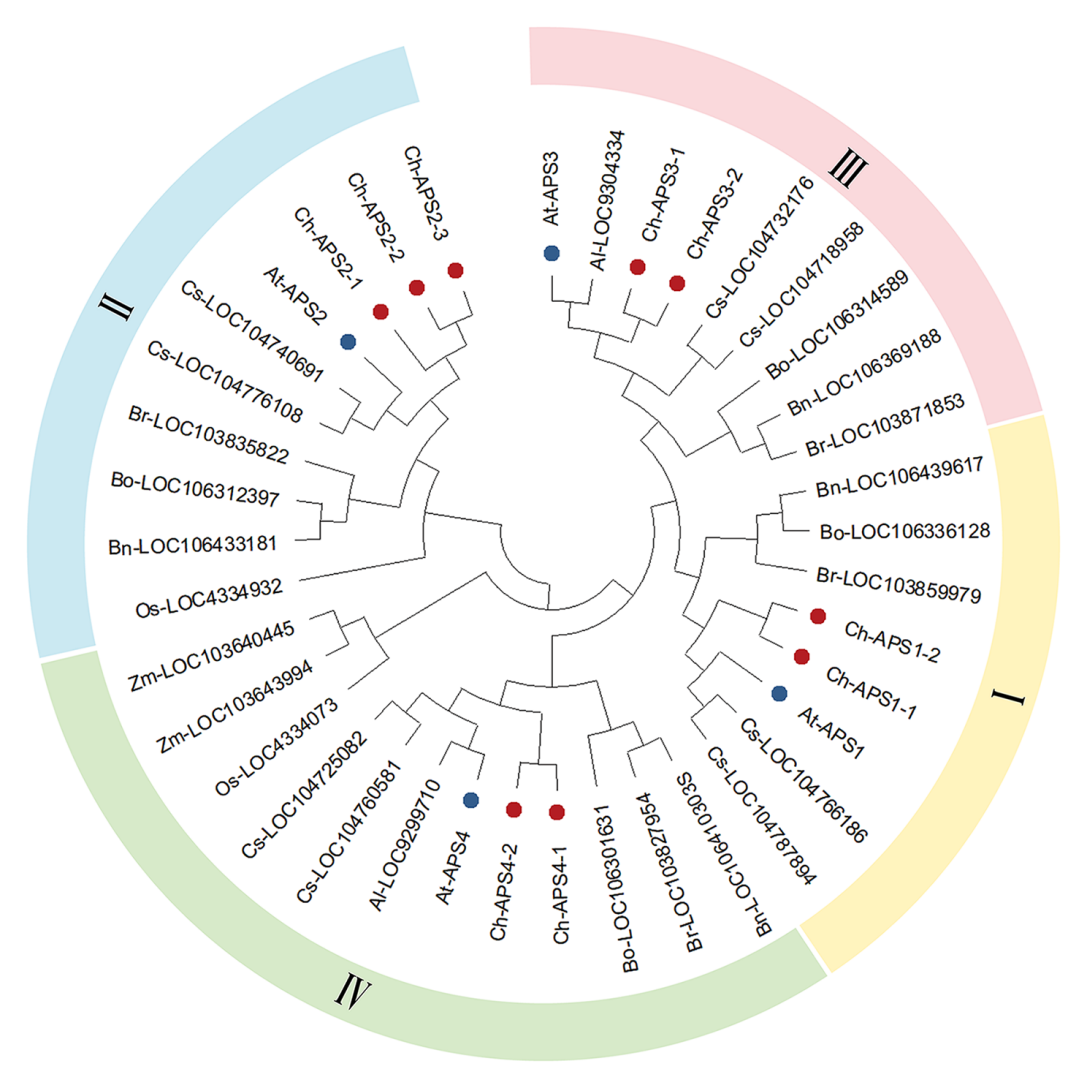
Phylogenetic relationships and structures of 39 *ATPS* genes. The phylogenetic tree of ATPS from *C. hupingshanensis* (Ch), *Arabidopsis thaliana* (At), *Camelina sativa* (Cs), *Brassica rapa* (Br), *Brassica napus* (Bn), *Brassica oleracea* (Bo), *Oryza sativa* (Os) and *Zea mays* (Zm). Colours represent different groups (Group I in yellow, Group II in blue, Group III in pink and Group IV in green). The genes ChATPSs (with red) and AtATPSs (with blue) are marked with dots of different colours

The protein sequences of 39 ATPSs were used, including 24 from monocotyledons, 4 from dicotyledons and 9 from *C. hupingshanensis*. We constructed a maximum likelihood (ML) phylogenetic tree by MEGA with default parameters (Fig. 2). We classified ATPS proteins into classes I, II, III, and IV based on bootstrap values and phylogenetic topology (Fig. 2).

Moreover, among the four categories, the second category is farther away from the other three categories, forming a relatively independent br*a*nch, suggesting that there may be functional differentiation between *ChATPS2* and *ChATPS1/3/4*. Among them, 3 out of 9 ChATPS genes were distributed in class II. *Arabidopsis thaliana*, *Brassica rapa*, *Brassica napus* and *Brassica oleracea* have only one ATPS gene in Class I, Class III and Class IV. However, *C. hupingshanensis* has two *ChATPS* genes in class I, class III and class IV.

### Analysis of the protein motif, conservative domain, Gene structure and sequence alignment of the ChATPSs

**Fig. 3:**
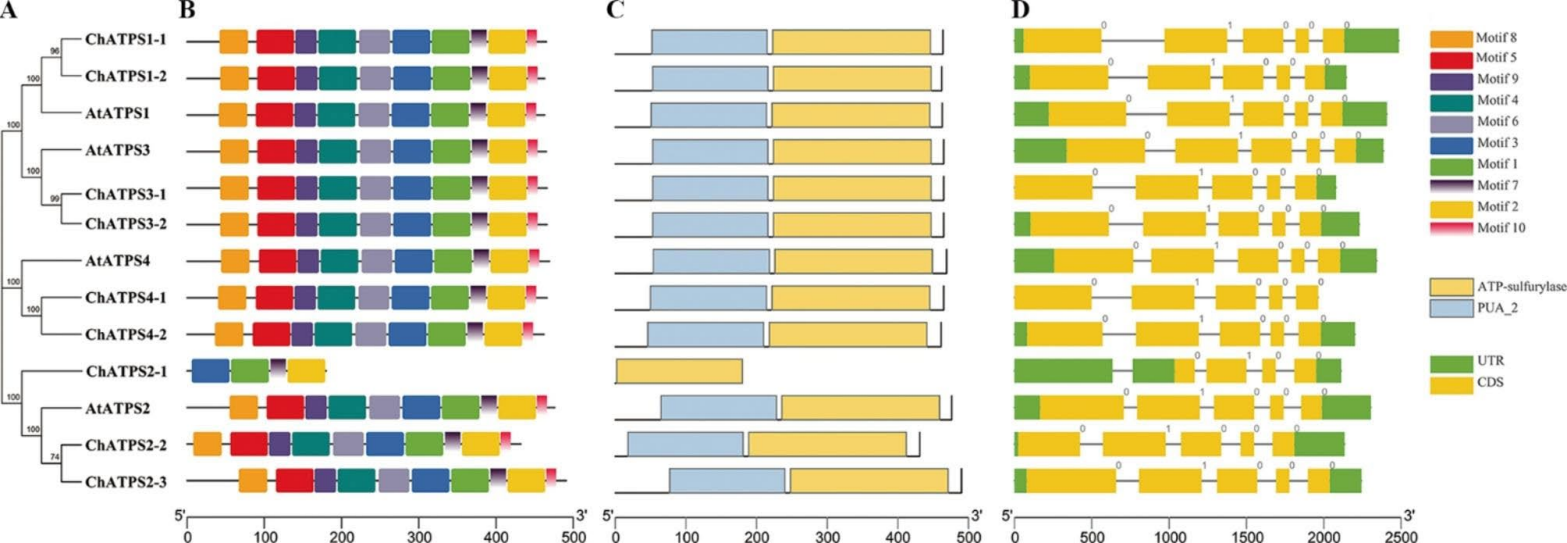
Phylogenetic trees, motifs, domains and gene structures of the ATPS gene family. **A **Phylogenetic tree of ATPS genes. **B-C** Conserved motifs and domains of the proteins; different colours represent different motifs or domains. **D** Exon-intron structures; exons are indicated by yellow boxes, and introns are indicated by lines

Genes have differentiated their regulatory and coding regions due to evolution usually based on gene duplication. As a result, amino acids may be replaced or altered, and the function of genes may be altered to suit different growth conditions. A simpler neighbour-joining phylogenetic tree was constructed from the ATPS protein sequences of *C. hupingshanensis* and *A. thaliana* to adequately recognize the protein motif, conserved domain and gene structure (Fig. 3).

On the basis of the annotated genome structure information, with the exception of ChATPS2-1, the homologous genes from different groups had the same number of introns/exons and a similar distribution. This result indicates that the ChATPS gene is extremely conserved in terms of structure and function.

The conserved motifs and conserved domains of AtATPS and ChATPS were analysed using the online software MEME and the server NCBI CDD to deeply explore the evolutionary relationship between members of different groups of *ChATPS*. Similar to the gene structure distribution results, the distribution of conserved motifs was conserved in different groups of genes. All ATPS contained all motif types, except for ChATPS2-1, in which motifs 4, 5, 6, 8, 9 and 10 were absent. While motifs 1, 2, 3 and 7 were correlated with the ATP-sulfurylase domain structure (PF01747), motifs 4, 5, 8 and 9 were associated with the PUA_2 (PF14306) domain structure.

### Conserved amino acid residues and chromosomal distribution and analysis of ChATPS genes

**Fig. 4:**
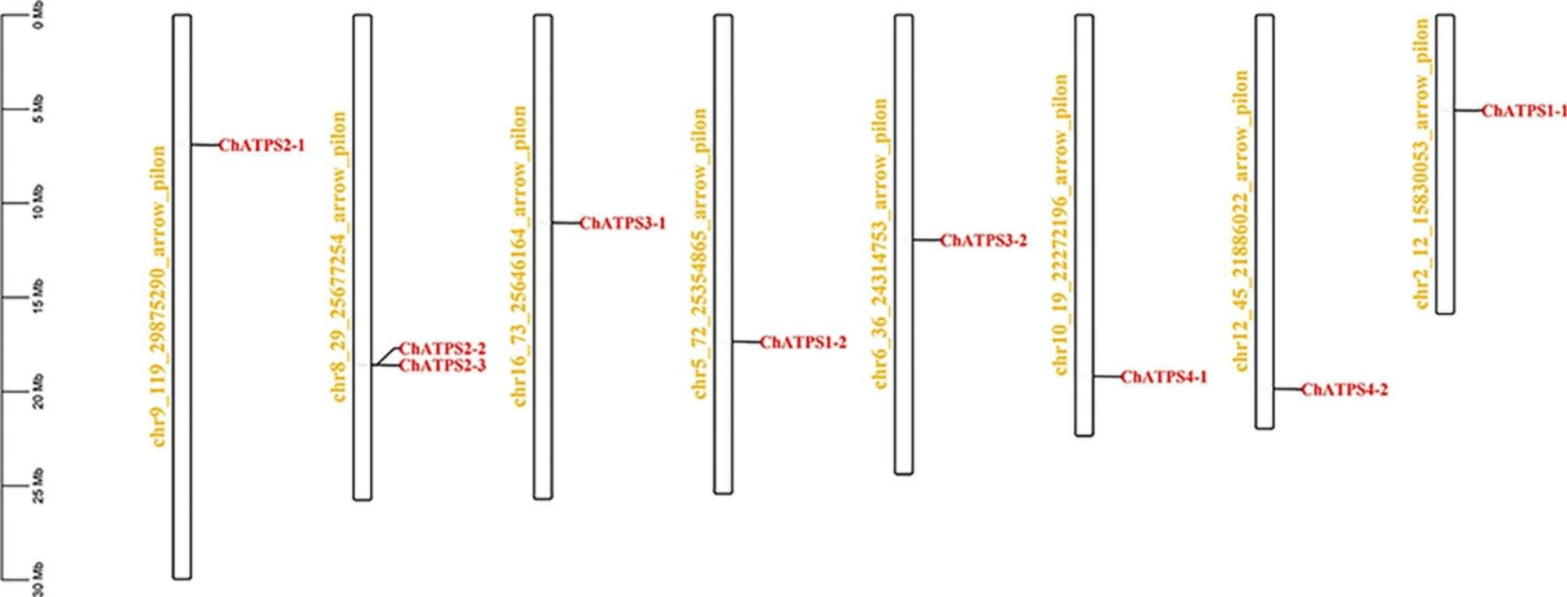
Chromosomal mapping of ATPS genes in *C. hupingshanensis*. The chromosome numbers are shown on the left side of each strip

The distribution of *ChATPS* genes on the 16 chromosomes of the *C. hupingshanensis* genome is relatively random (Fig. 4). With the exception of chromosome 8, which contains two *ChATPS* genes (*ChATPS2-2* and *ChATPS2-3*), each of the remaining *ChATPS* genes is located on a separate chromosome. Notably, most of the *ChATPS* genes are located at the distal end of the chromosome, with five members in a reverse distribution and the other four members in a positive distribution.

From the alignment of the full-length sequences of ChATPS proteins (Fig. S1), the ChATPS protein displayed an N-terminal mature peptide (^50^GLIEPDGKLVDLVVPEPRR^69^), which was characterized by transit peptide localization to the chloroplast and had greater than 60% N-terminal homology with the native chloroplast ATPS of *Arabidopsis* and *spinach*[40, 46, 47].

**Fig. 5:**
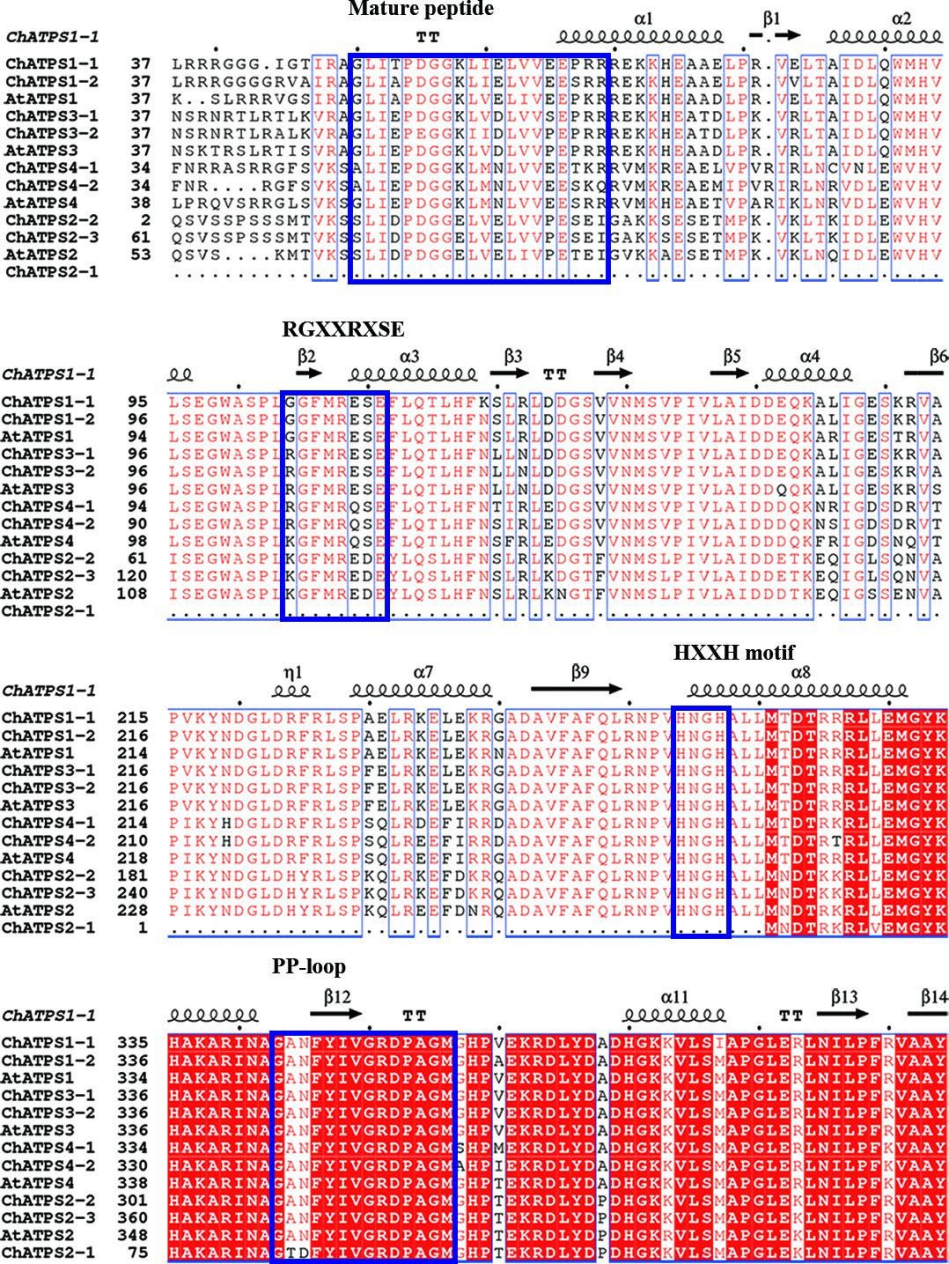
Multiple alignment of partial sequences of the *C. hupingshanensis* ATPS proteins. Secondary structure elements are defined according to ESPript3.0 [50]. The helixes represent alpha helices, and the arrows represent beta strands

The relatively conserved sequence exists in the C-terminus (Fig. 5). We further analysed the conservation of amino acid residues in this domain, similar to the analysis in *A. thaliana*. The amino acid residues in the C-terminal domain remained conserved at most loci, which was assumed to be required for ATP sulfurylase. Remarkably, two conserved motifs present in the ChATPSs are the PP-loop (^343^GANFYIVGRDPAGM^360^) and HXXH motif (^254^HNGH^257^), except for ChATPS2-1.

Furthermore, cysteine residues are key redox targets and play important roles in redox regulatory mechanisms [48]. According to Prioretti et al. [49], algal ATPS proteins contain a large number of cysteine residues and are highly conserved compared to ATPS genes of plants and other organisms. Their research showed that cyanobacteria, marine cyanobacteria, green algae, hyaluronicum and heteroalgae contain five highly conserved cysteine residues. However, the cysteine residues identified in our study are not conserved structures and are few in number. These data are inconsistent with previous findings [49].

Combined with the gene structure, conserved domains, motifs and multiple sequence alignment results, we speculate that ChATPS2-1 may be a mutation or functional redundancy during evolution. Therefore, we will not perform protein function exploration and gene expression analysis.

### Prediction of secondary and tertiary structures of ATPS protein

**Fig. 6:**
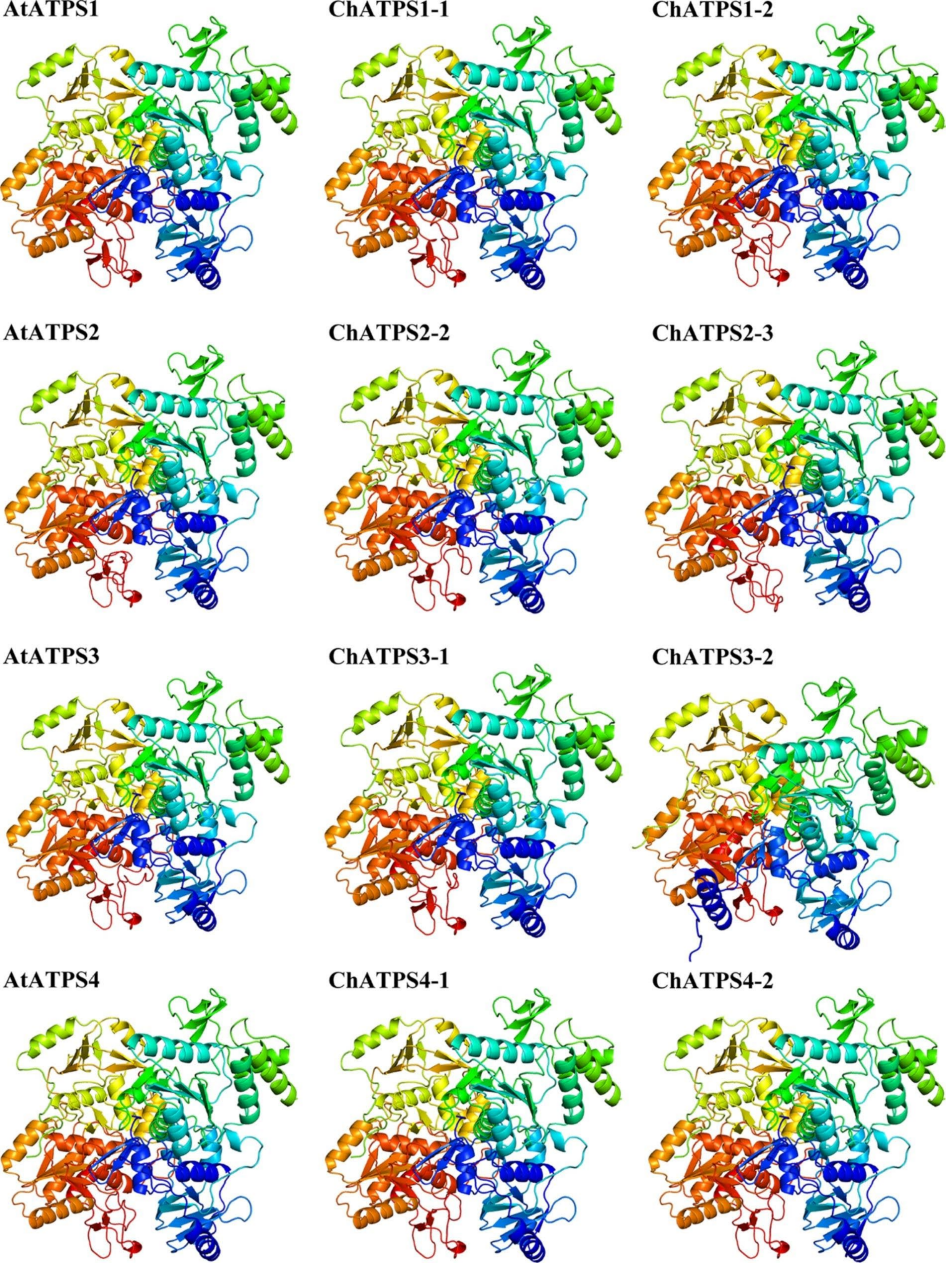
Predicted 3D structures of ChATPSs and AtATPSs by the SWISS-MODEL server

Secondary structure analysis of the eight ChATPS proteins (Table S3) revealed the presence of α-helices (29.89–32.04%), extended strands (15.70–19.39%), β-turns (5.51–9.44%) and random coils (38.75–46.67%), indicating minor structural differences in ChATPS sequences. BLAST searches were performed on the SWISS-MODEL library to determine a suitable template for the *C. hupingshanensis* ATP sulfurylase. The 3D structures of ChATPS and AtATPS used for docking were also predicted by the SWISS-MODEL server (Fig. 6). The soybean ATP sulfurylase (PDB code: 4MAF) with the highest similarity scores (ranging from 76.62 to 86.07%) was selected as a template (Table 2). The 3D modelled protein structures of ChATPS have high GMQE (0.77 ~ 0.89) and QMEAN (0.87 ~ 0.89) scores, indicating high confidence in the modelled structures. These ChATPS models were validated with the Structural Analysis and Validation Server (SAVES). Ramachandran plots show that nearly 90% of all models have residues in the favourable region, with ≥ 95% of residues in the core and allowable regions, which is sufficient to indicate the reliability of the 3D model. Overall quality factor values were greater than 90 in all generated models. The average 3D-1D scores for the nine model residues were higher than 0.2. Furthermore, the plausibility of torsion angles and covalent geometric distributions within the model are indicated by G-factor values, all greater than − 0.5. In general, homology models are stable and reliable. ProSA analysis showed that all models had Z scores between − 10.99 and − 11.65. Finally, an LG score (greater than 3) indicates that the protein model is of good quality. All ChATPS protein structures achieved significant scores, with LGscores greater than 7.3. These results indicate that these models obtained with the homology model are acceptable and can be used for further studies.

**Table 2:** Validation of the modelled structures of ChATPS

Gene name	Template	Sequence Identity	Coverage	GMQE	QMEAN	Verify 3D	ERRAT	G-factors			Z Score		LGscore		MaxSub		
								**Dihedrals**	**Covalent**	**Overall**							
*ChATPS1-1*	4maf.1. A	86.07%	0.87	0.84	0.9	92.37%	95.103	-0.22	0.06	-0.1	-11.23	9.201				-0.453	
*ChATPS1-2*	4maf.1. A	85.57%	0.87	0.84	0.89	90.49%	95.466	-0.23	0.07	-0.1	-11.01	9.257				-0.453	
*ChATPS2-2*	4maf.1. A	76.87%	0.93	0.89	0.87	94.74%	95.288	-0.22	0.06	-0.1	-11.49	9.056				-0.458	
*ChATPS2-3*	4maf.1. A	76.62%	0.82	0.77	0.87	96.50%	95.413	-0.22	0.07	-0.1	-11.53	9.12				-0.462	
*ChATPS3-1*	4maf.1. A	85.32%	0.86	0.83	0.89	91.99%	94.401	-0.26	0	-0.14	-11.32	9.282				-0.438	
*ChATPS3-2*	4maf.1. A	84.33%	0.86	0.83	0.88	87.13%	91.932	0.05	-0.09	0.01	-10.99	7.305				-0.338	
*ChATPS4-1*	4maf.1. A	83.33%	0,86	0.83	0.89	93.76%	96.525	-0.23	0.07	-0.1	-11.55	9.1				-0.462	
*ChATPS4-2*	4maf.1. A	83.03%	0.87	0.83	0.89	92.73%	96.778	-0.22	0.06	-0.1	-11.65	9.125				-0.463	

### Molecular Docking

**Fig. 7:**
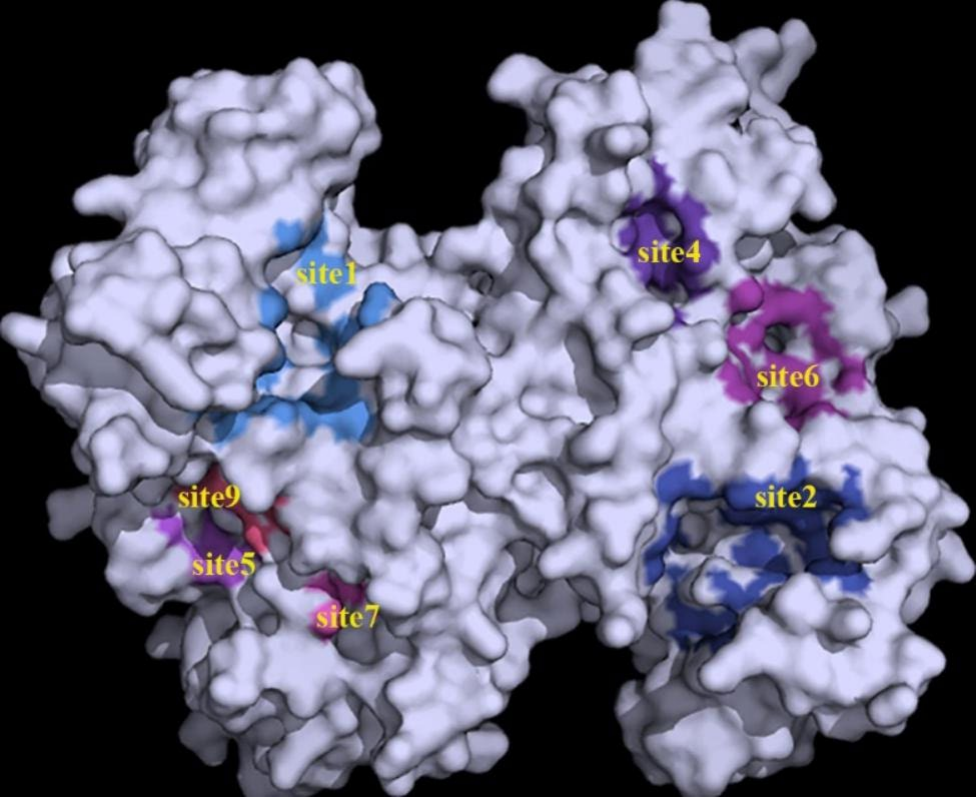
Visualization of some of the predicted ligand-binding sites for protein ChATPS1-2 by Prankweb

Molecular docking is a novel technique for identifying binding modes or forces of ligand-protein complexes and is widely used in structural molecular biology and drug discovery [51]. First, we used Prankweb to predict and visualize the ligand-binding sites of ATPSs [52]. Several ligand-binding sites were predicted for each ATPS, ranging from 10 to 15 for ChATPSs and 7 to 13 for AtATPSs. The online server numbers each site with Arabic numerals starting from 1 according to the calculated probability score, which is also the basis for our naming of binding sites. Therefore, for different proteins, the same Arabic numerals do not necessarily represent the same spatial position in each protein. For example, part of the ligand binding sites of protein ChATPS1-2 are visualized in Fig. 7. Afterwards, protein-ligand docking and molecular simulations were performed using the AutoDock Vina program [53]. The binding energy of protein-ligand docking, which is an important criterion for interaction, was recorded, with a lower binding energy being considered more stable [53]. The structure of protein-ligand interactions was finally analysed using a protein-ligand interaction analyser (PLIP) and visualized with PyMOL [54, 55].

**Fig. 8:**
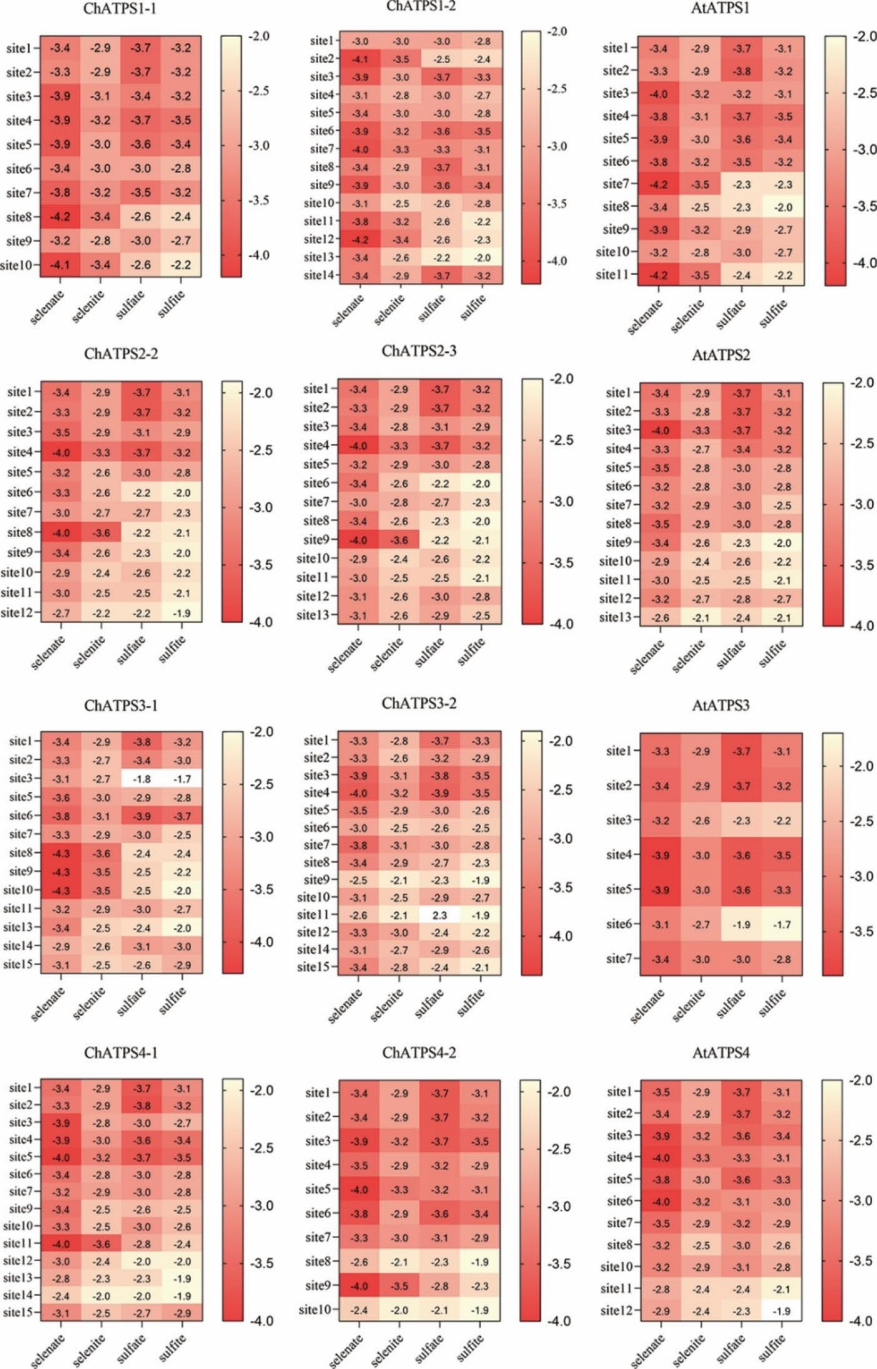
The binding energy of compounds with all the ligand-binding sites of ATPS (ChATPS and AtATPS). The bottom of the heatmap represents four different ligands, and the ordinate represents the ligand-binding site predicted by Prankweb for each protein. The value represents the binding energy displayed by the ligand and the binary ATP-ATPS docking complex, unit: kcal mol^-1^

Bioinformatics analysis and preliminary study were used to verify the binding ability of selenate, selenite, sulfate, and sulfite with ChATPS according to network analysis and preliminary research. The docking binding energy of each ligand compound to the protein molecule is displayed in the heatmap (Fig. 8). When comparing the docking results between the ligand and ChATPS, the most noticeable difference was the interaction energy, which ranged from − 4.3 to 2.3 kcal mol^-1^. We found that all ChATPS had stronger affinity for selenate than other compounds, with ChATPS1-1 (-4.2 kcal mol^-1^), ChATPS1-2 (-4.2 kcal mol^-1^) and ChATPS3-1 (-4.3 kcal mol^-1^) showing stronger affinity for selenate than other genes.

**Fig. 9:**
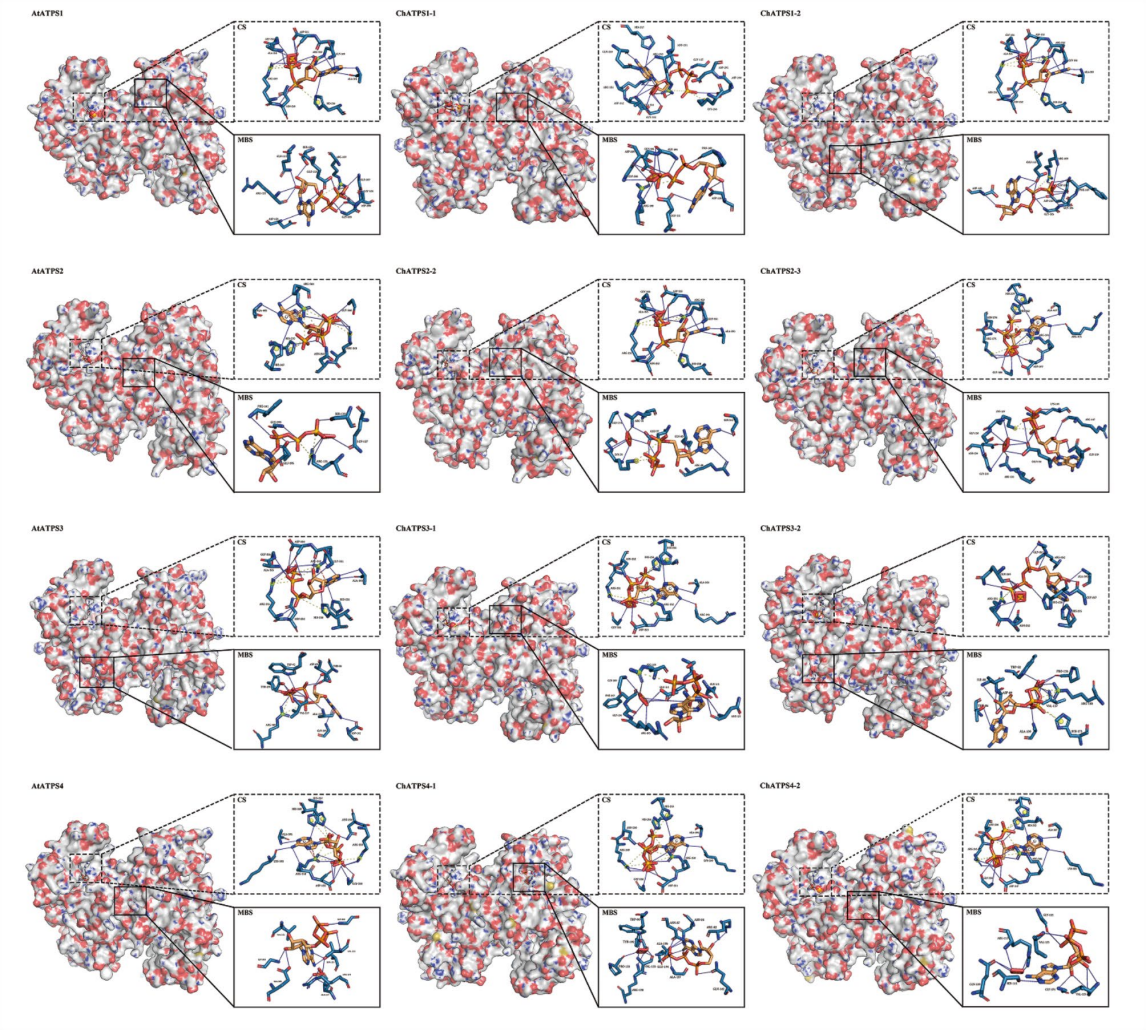
Interactions of the binary ATP-ATPS complex with selenate. The left panel is the overall view, and the right panel is the focused view. The ATPS protein is shown on the surface, the amino acid residues at the binding site are grey-blue, and the ligand (ATP and selenate) is heavy yellow. The blue solid line represents a hydrogen bond, the yellow dashed line represents a salt bridge, and the red dashed line represents a π-cation interaction. CS: putative binding mode of ATP and selenate to model the ATPS protein structure at the catalytic site. MBS: ATP and selenate are in a putative binding mode that mimics the protein structure of ATPS at the site of minimum binding energy, the site of maximum affinity binding

When analysing protein-ligand interactions, it was found that most of the amino acids at site 1 of each protein consisted of two conserved motifs, ATPS, PP-loop and HXXH. It shares a similar spatial structure with the ATPS catalytic site [43, 56]. We call site 1 of each protein the catalytic site, which is abbreviated as CS in Fig. 9. Therefore, we selected the catalytic site and one of the binding sites with the minimum binding energy in the docking simulation to visualize the interaction of the binary ATP-ATPS complex with selenate, including the amino acid residues involved in the interaction and the interaction forces (hydrogen bonds, salt bridges and π-cation interactions). Hydrogen-bond interactions were found to be necessary for the interactions of the binary ATP-ATPS complex with selenate.

The interaction at the catalytic site is similar to GmATPS in that the ligands are surrounded by positively charged residues Arg250, His254, His257 and Arg350. In previous studies, most of these conserved amino acid residues were shown to interact with the β- or γ-phosphates of ATP [43]. In our study, these conserved ATP residues still interact with ATP phosphate and hydrogen bond with ATP adenine and selenate. These may be the reasons for the higher affinity of ChATPS for selenate.

At the maximum affinity binding site, similar to the catalytic site, the ligand is surrounded by positively charged amino acids Arg105 and Arg109. Although not among the characteristic catalytic sites of ATPSs, most of the highest affinity sites in ChATPS are located in similar spatial positions and have similar amino acid compositions (Arg105, Gly106, Arg109, Ser111 and Glu112).

### Expression profiles of ATPS genes in different tissues under Se stress

ATP-sulfurylase can participate in plant responses to several abiotic stresses through different sulfides. To better understand the molecular functions of *ChATPS* genes under abiotic stress conditions, RT-qPCR technology was used to analyse the expression of nine *ChATPS* genes in *C. hupingshanensis* leaves under different concentrations of Se stress (0 µg Se L^-1^, 100 µg Se L^-1^ and 80,000 µg Se L^-1^).

**Fig. 10:**
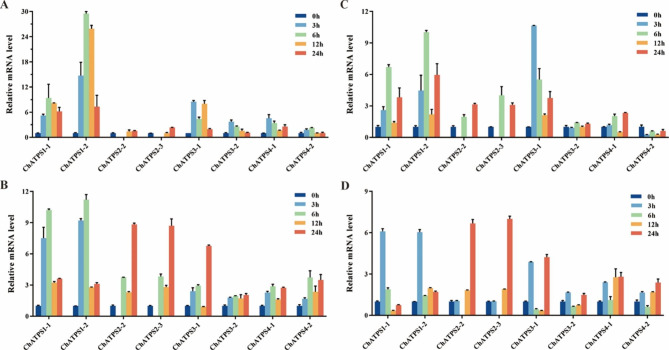
Expression of the *ChATPS* gene in different concentrations of selenium stress and different tissues. **A** Expression of ChATPS family genes in roots under low-concentration selenium stress (100 µg Se L^-1^). **B** Expression of *ChATPS* family genes in leaves under low-concentration selenium stress (100 µg Se L^-1^). **C** Expression of *ChATPS* family genes in roots under high-selenium stress (80,000 µg Se L^-1^). **D** Expression of *ChATPS* family genes in leaves under high-selenium stress (80,000 µg Se L^-1^). The abscissa represents 9 *ChATPS* genes, and the ordinate represents different time points (0 (control group), 3, 6, 12 and 24 h) under different treatments (0 µg Se L^-1^, 100 µg Se L^-1^ and 80,000 µg Se L^-1^) relative expression levels. Samples at 0, 3, 6, 12 and 24 h are represented by dark blue, light blue, green, orange and red, respectively. Each data point represents the mean ± standard deviation (SD) (n = 3). Error bars represent the standard deviation

Among the *ChATPS* family genes measured by qRT-PCR, under low-concentration selenium stress (100 µg Se L^-1^), the gene expression in roots was upregulated (Fig. 10 A). Among them, *ChATPS1-2* genes were highly upregulated (approximately 29.5-fold) at 6 h. *ChATPS1-1* was upregulated approximately 9.4-fold at 6 h, and *ChATPS3-1* was upregulated approximately 8.5-fold at 3 h. The upregulation of the remaining six genes in roots was relatively small (1.5- to 4.6-fold) under low-concentration selenium stress. The expression of *ChATPS* family genes was upregulated in leaves under low-concentration selenium stress (Fig. 10B). The upregulation of *ChATPS1-1* and *ChATPS1-2* gene expression was prominent (approximately 10.2-fold and 11.2-fold) at 6 h. *ChATPS2-2*, *ChATPS2-3* and *ChATPS3-1* were upregulated at 24 h by approximately 8.8-fold, 8.7-fold and 6.8-fold, respectively. The remaining four genes were upregulated to a smaller extent (2 to 3.7 times) in leaf parts under low-concentration selenium stress.

Under high-selenium stress (80,000 µg Se L^-1^), only *ChATPS4-2* was downregulated in roots. The expression of other members of the *ChATPS* gene family was upregulated (Fig. 10 C). Among them, *ChATPS1-1* and *ChATPS1-2* were upregulated approximately 6.7-fold and 10-fold at 6 h, respectively, and *ChATPS3-1* was upregulated approximately 10.6-fold at 3 h. The upregulation of the remaining four genes in roots was relatively small (1.4- to 4-fold). *ChATPS* family members were upregulated to varying degrees in leaves under high-concentration selenium stress (Fig. 10D). Simultaneously, *ChATPS1-1* and *ChATPS1-2* were upregulated approximately 6.1-fold and 6-fold at 3 h, respectively. *ChATPS2-2* and *ChATPS2-3* were upregulated approximately 6.7-fold and 7-fold at 24 h, respectively. The remaining four genes were upregulated to a lesser extent (1.6- to 4.2-fold) in leaf parts under high-concentration selenium stress.

Based on these data, *ChATPS* may play an important role in selenium detoxification by promoting selenide production.

## Discussion

In our study, more *C. hupingshanensis ATPS* genes were identified from the genome database using bioinformatics methods, which is a potential reason for Se tolerance and Se accumulation capacity in Se hyperaccumulators. In total, 9 *ATPS* genes were recognized in *C. hupingshanensis*, which is relatively more abundant than that of *Arabidopsis thaliana* (4 *AtATPS*), *Brachypodium distachyon* (2 *BdATPS*), *Cucumis sativus* (3 *CsATPS*), *Oryza sativa* (2 *OsATPS*), *Phaseolus vulgaris* (2 *PvATPS*), *Prunus persica* (2 *PpATPS*), *Sorghum bicolor* (2 *SbATPS*), *Solanum lycopersicum* (3 *SlATPS*), *Brassica rapa* (6 *BrATPS)*, *Vitis vinifera* (2 *VvATPS*), and *Daucus carota* (3 *DcATPS*) [38]. Among them, ChATPS1-2 is shorter than other genes and lacks the motif of ATPS feature. We did not perform functional analysis or gene expression analysis. However, there are still few studies on the interaction mechanism between ATP sulfurase and selenite or selenite. Using molecular docking technology to model the interaction between ATP sulfurase and selenate model compounds, we observed that Ch*ATPS* bound with the greatest efficiency to selenate, while its bond with sulfite was weaker. In addition, we found that the binding orientation of the ligand model compounds inside ATPS varied greatly. The site of minimum binding energy is the same for selenate and selenite and different from that of sulfate (Table S5 and S6). This indicates that selenium hyperaccumulators appear to be able to differentiate between sulfate and selenate uptake and to preferentially accumulate selenium over sulfur, which is consistent with the experimental results of Schiavon et al. [57].

To better understand the molecular functions of *ATPS* genes under selenium stress, RT-qPCR was used to analyse the expression of eight *ChATPS* genes in leaves and roots of *C. hupingshanensis* under different concentrations of selenium (Fig. 10). Under the low selenium concentration treatment, the expression level of *ChATPS1-2* in roots was upregulated by approximately 29-fold, which was significantly higher than that in the roots of the high concentration of selenium treatment (approximately 10 times), which had a certain stress effect on *C. hupingshanensis*. Additionally, the *ChATPS1-2* gene also has a strong affinity for sodium selenate, which catalyses the reaction under suitable growth conditions of selenium concentration, accelerates the metabolism of selenium, and shows a strong ability to accumulate selenium. We think that the low expression level of genes with strong affinity prevents excess selenium from entering the metabolic pathway, thereby resisting the stress of high concentrations of selenium and showing excellent selenium tolerance. We speculate that *C. hupingshanensis* evolved more ATPS gene members than *Arabidopsis* and other higher plants to adapt to the high-selenium environment. This phenomenon deserves our attention in future explorations of the mechanism. Additionally, the upregulation of ChATPS1-2 gene expression was significantly higher than that of other members, and the affinity of ChATPS1-2 protein with the substrate was also stronger in the molecular docking simulation exploration. Based on the above two points, we will select the ChATPS1-2 gene for further research.

The ATPS family genes of *C. hupingshanensis* shared conserved structures and motifs but had a stronger affinity for selenate. This allows inorganic selenium to enter metabolic pathways faster, helping plants accumulate selenium. First, ChATPSs exhibited high similarity to AtATPSs in motifs, ATPS-type domains, CDS regions, and exons (Fig. 3B), which makes them persist in catalytic function. Based on their interfamily similarities, relation with their homologues from other species, such as *Arabidopsis thaliana*, and motif distribution, the ChATPS gene family was classified into four subfamilies. The obtained results are in line with those of previously reported studies in *Arabidopsis*. Moreover, the conserved MEME motifs of ATPS proteins also exhibited corresponding permutation and combination with their phylogenetic relationship (Fig. 3 C). These results implied the possibility that the ATPS gene family may function in a conserved manner in *C. hupingshanensis* and *Arabidopsis*. Analysis of the characteristics of the identified ATPS proteins showed that all ATPSs have no signal peptides and no transmembrane region. The absence of a signal peptide, transmembrane domain and cysteine residues and disulfide bonds indicate that these proteins are likely intracellular in nature. In addition, according to the conserved amino acid residue analysis, two conserved domains present in ChATPS are the PP-loop and HXXH motif (Fig. 5) [44, 56, 58], which constitutes Site1 of these proteins. In addition, the results of molecular docking calculated by computer algorithms cannot completely simulate the actual conformational changes of proteins. Therefore, when we docked the protein as a semiflexible molecule to the substrate, the optimal binding region did not appear in the catalytic domain. We are still conducting further biological experiments to explore the true binding form. On the other hand, after molecular docking simulations, it was found that the ligand selenate has a stronger affinity for the ChATPS protein than AtATPS. In terms of amino acid composition at the affinity site, hydrogen bonds, salt bridges and π-cation interactions together form affinity interactions. Additionally, we found that the sites with higher affinity in the ChATPS protein were enriched in the following residues: Arg105, Gly106, Arg109, Ser111 and Glu112. These residues constitute a relatively conserved sequence ^105^RGXXRXSE^112^ in the ChATPS genes (Fig. 5).

In conclusion, the results of this study provide important insights into the function of ChATPS genes in Se hyperaccumulators and their responses to selenium stress conditions. In this context, nucleotide and protein sequence analysis and phylogeny, determination of gene expression profiles of *C. hupingshanensis* under selenium stress, and 3D structure prediction of ChATPS was performed. Notably, the ATPS gene is highly conserved. In addition, the *C. hupingshanensis* ATPS gene showed different expression patterns according to time and stress intensity, indicating dynamic regulation. The results of this study may support the understanding of the selenium assimilation pathway in higher plants under abiotic stress conditions.

## Materials and methods

### Genome-wide identification of ATPS Family genes

*C. hupingshanensis* genome and its annotation file were obtained from the Genome Warehouse BIG Data Center under accession number PRJCA005533. To identify the ATPS gene family in *C. hupingshanensis*, the AtATPS protein sequences were downloaded from the *Arabidopsis* Information Resources (TAIR) database (https://www.arabidopsis.org/). Using AtATPS as the query sequence, the most representative ChATPS protein sequence was extracted by the Blast Zone of TBtools software [59]. In addition, the extracted ChATPS proteins were further checked by NCBI BLAST (https://blast.ncbi.nlm.nih.gov/blast/Blast.cgi). The conserved domains of ChATPS proteins were analysed by CD-search (https://www.ncbi.nlm.nih.gov/Structure/cdd/wrpsb.cgi).

### Bioinformatic analysis of the ATPS genes

ChATPS chromosomal location information was extracted from the *C. hupingshanensis* genome GFF file and plotted by “Gene Location Visualize from GTF/GFF” of TBtools software. In addition, the molecular weight (MW), isoelectric point (pI) and other physical and chemical properties of the ChATPS family can be predicted and analysed using the online tool ExPASy (https://web.expasy.org/protparam/) [60]. The online software SignalP 5.0 (http://www.cbs.dtu.dk/services/SignalP/) was used to predict signal peptides. WoLF PSORT (https://wolfpsort.hgc.jp/) was used for ChATPS gene subcellular localization predictions. The transmembrane regions of proteins were analysed by TMHMM 2.0 (http://www.cbs.dtu.dk/services/TMHMM/) [61]. The AtATPS and ChATPS protein sequences were aligned by ClustalW (https://www.genome.jp/tools-bin/clustalw), and the alignment result was further processed by ESPript 3.0 (https://espript.ibcp.fr/ESPript/cgi-bin/ESPript.cgi) to output the image [50].

Submit the ChATPS and AtATPS protein sequences to perform a conserved motif scan on the MEME website (http://meme-suite.org/tools/meme) with the MEME-motif set to 10. ChATPS and AtATPS protein sequences were submitted to CDD: NCBI’s conserved domain database (https://www.ncbi.nlm.nih.gov/Structure/bwrpsb/bwrpsb.cgi) to obtain conserved domain information. The intron-exon gene structure information of the *ChATPS* and *AtATPS* genes was extracted from the GFF files of the respective genomes. Submit the Newick Tree file output by MEGA, the xml file obtained from the MEME website, the HitDate file and genome GFF file obtained by NCBI-CDD, and visualize by “Gene Structure View (advanced)” of TBtools.

### Phylogenetic analysis of ChATPS

To explore the phylogenetic relationship of *ChATPS* family genes, 4 from *Arabidopsis thaliana* (At), 8 from *Camelina sativa* (Cs), 4 from *Brassica rapa* (Br), 4 from *Brassica napus* (Bn), 4 from *Brassica oleracea* (Bo), 2 from *Oryza sativa* (Os) and 2 from *Zea mays* (Zm) were downloaded from NCBI for multiple sequence alignment. The amino acid sequences were aligned using Clustal W, and then a maximum likelihood (ML) tree was constructed with all of the ChATPS protein sequences using MEGA 11, bootstrap = 1000 repetitions.

### Homology modelling and validation of ChATPS

SOPMA was used to predict the protein secondary structure [62]. Then, we searched and selected the best crystal structure as a template in the SWISS-MODEL (https://swissmodel.expasy.org/) template library and used the SWISS-MODEL web server to model the ChATPS protein homology. The final 3D models of ChATPS were validated using the online server SAVES 5.0 (https://servicesn.mbi.ucla.edu/SAVES/) with various functions.

### Ligand Preparation and Molecular Docking

The compounds (ATP, selenate, selenite, sulfate, sulfite) used in this study were selected from the Chemspider database. Their structures were sketched with ChemSketch saved in protein data bank format. PrankWeb [52] was used to predict protein active sites.

Experiments with the docking of proteins and ATP sulfurylase were performed using AutoDock v4.2 and AutoDock Vina v1.1.2 [53]. We used AutoDock v4.2 to modify proteins and ligand compounds, adding all hydrogens, incorporating nonpolar hydrogens and calculating Gasteiger charges. We used AutoDock Vina to perform the molecular docking of compounds with ATP sulfurylase proteins with the exhaustiveness setting at 10. The best aptamer conformations are selected based on their minimal binding energies.

First, the ATP-Protein complex PDB file was formed by docking the substrate ATP with the binding pockets of each protein molecule by AutoDock Vina and PyMOL. Only pockets with negative binding energy can be docked in the next step. Then, the small molecule ligand and ATP-protein complex PDB files were docked by AutoDock Vina to form a small molecule-ATP-protein complex PDB file. The interaction of the small molecule-ATP-protein complex (hydrogen bond and hydrophobic interaction) was analysed and visualized by PLIP and PyMol, and the docking binding energy was visually analysed by GraphPad Prism version 9.0.0 for Windows, GraphPad Software, San Diego, California USA, www.graphpad.com.

### Gene expression analysis

Seeds of *C. hupingshanensis* were collected from the Yutangba Colour Mine in Enshi, Hubei Province, China. Plants were placed in a room with a constant temperature of 22 ± 1 °C, a photoperiod of 16 h, and an irradiance of 1500 mol^− 2^ ms^− 1^. Thirty-nine seedlings approximately 10 cm tall and four months old were selected, and the roots were rinsed with vermiculite and equilibrated in Hoagland’s solution for two days. The samples were treated with selenium at different concentrations (0 µg Se L^-1^, 100 µg Se L^-1^, 80,000 µg Se L^-1^), the actual concentration of elemental selenium coming from the selenite (analytical reagent, Sinopharm Chemical Reagent Co., Ltd, Shanghai, China), and the samples treated with 0 µg Se L^-1^ were used as experimental controls. Leaves and roots of 3 seedlings were isolated from each treatment at 0 h, 3 h, 6 h, 12 and 24 h, and these samples were snap frozen in liquid nitrogen for RNA extraction.

Total RNA was extracted from root and leaf samples by the TRIzol method, and the RNA concentration and quality were detected by a NanoDrop 2000. RNA integrity and genomic DNA contamination were detected by gel electrophoresis. RNA samples were treated with RNase-Free DNase to remove residual genomic DNA. Real-time PCR was performed on ABI StepOne Plus. The primers used in qRT-PCR analysis for ChATPS are shown in Table S4. The EvoScript RNA SYBR Green I Master Kit (Roche) was used to quantitatively detect the expression of target genes in the samples, and the ΔΔ-CT method was used to calculate the gene expression. Column graphs were generated using GraphPad Prism version 9.0.0 for Windows, GraphPad Software, San Diego, California USA, www.graphpad.comprism. All assays were carried out in triplicate.

## Electronic supplementary material

Below is the link to the electronic supplementary material.

Supplementary Figure S1: Multiple sequence alignment of all the ChATPS proteins; Supplementary Table S1: Physicochemical properties of ATPS proteins in *C. hupingshanensis*. Supplementary Table S2: The gene coding sequences and protein sequences of ChATPS. Supplementary Table S3: Secondary structure analysis of the nine proteins. Supplementary Table S4: Primers used in qRT-PCR analysis for ChATPS. Table S5: The binding energy of each ligand to each protein at the catalytic site (unit: kcal mol^-1^). Table S6: At the minimum binding energy site of selenate and protein, the binding energy of each ligand and protein (unit: kcal mol^-1^).

Supplementary Material 2

## Data Availability

The data that support the findings of this study are available from the corresponding author upon reasonable request.
