# Supplementary figures and images for: Gene Identification, expression analysis and molecular docking of ATP sulfurylase in the selenization pathway of Cardamine hupingshanensis
Source: BMC Plant Biol. 2022 Oct 18;22:491. doi: 10.1186/s12870-022-03872-7 (PMC9578213; doi:10.1186/s12870-022-03872-7)

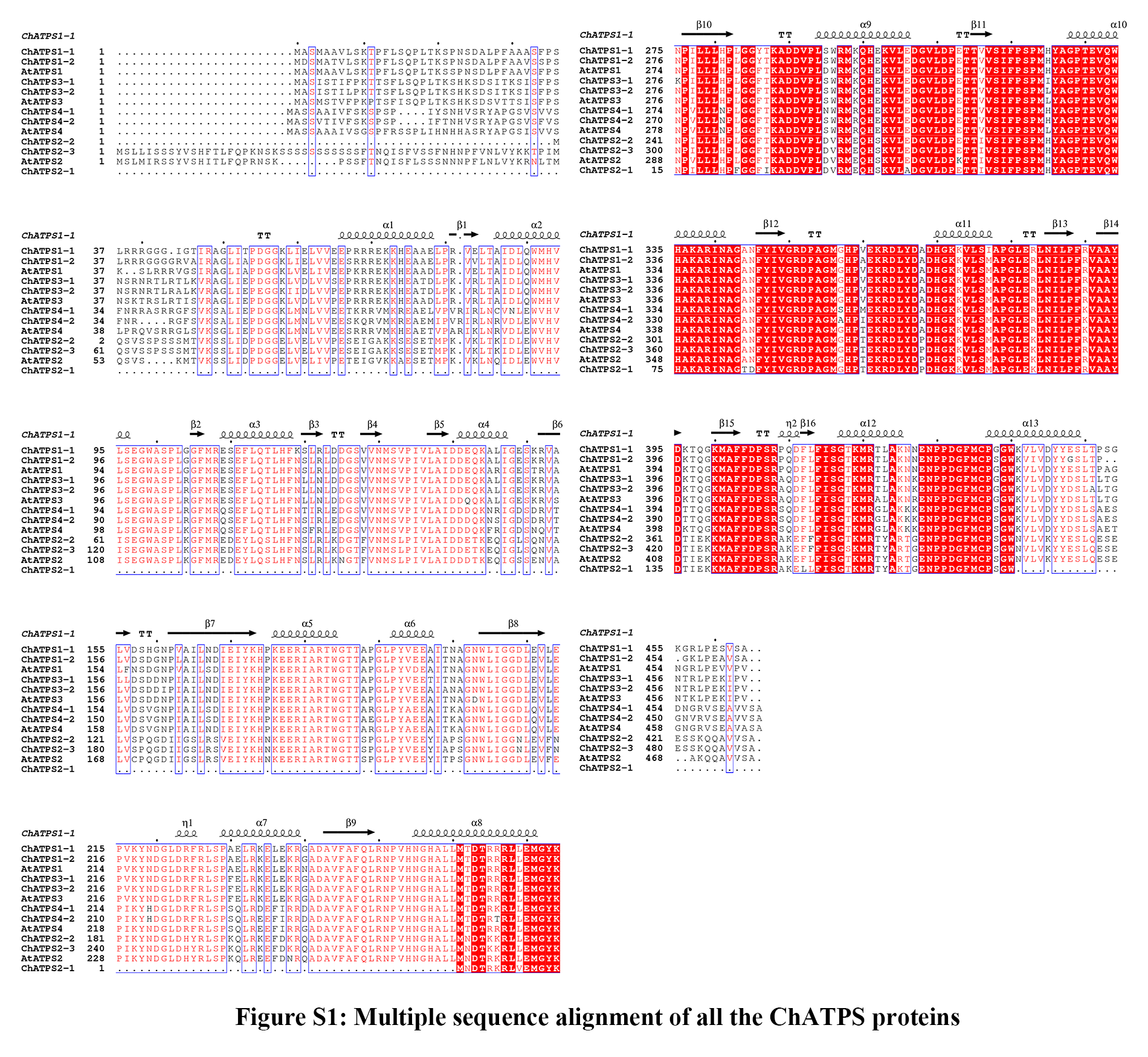

Supplement: Supplementary file 2 — Supplementary Material 2 [file 12870_2022_3872_MOESM2_ESM.png]
